# Association of blood pressure variability during acute care hospitalization and incident dementia

**DOI:** 10.3389/fneur.2023.1085885

**Published:** 2023-02-07

**Authors:** Joseph E. Ebinger, Matthew P. Driver, Patrick Botting, Minhao Wang, Susan Cheng, Zaldy S. Tan

**Affiliations:** ^1^Department of Cardiology, Smidt Heart Institute, Cedars-Sinai Medical Center, Los Angeles, CA, United States; ^2^Department of Neurology and Medicine, Cedars-Sinai Medical Center, Los Angeles, CA, United States; ^3^David Geffen School of Medicine at UCLA, Los Angeles, CA, United States

**Keywords:** dementia, blood pressure variability, hypertension, hospitalization, risk prediction

## Abstract

**Background and objectives:**

Recognized as a potential risk factor for Alzheimer's disease and related dementias (ADRD), blood pressure variability (BPV) could be leveraged to facilitate identification of at-risk individuals at a population level. Granular BPV data are available during acute care hospitalization periods for potentially high-risk patients, but the incident ADRD risk association with BPV measured in this setting is unknown. Our objective was to evaluate the relation of BPV, measured during acute care hospitalization, and incidence of ADRD.

**Methods:**

We retrospectively studied adults, without a prior ADRD diagnosis, who were admitted to a large quaternary care medical center in Southern California between January 1, 2013 and December 31, 2019. For all patients, determined BPV, calculated as variability independent of the mean (VIM), using blood pressure readings obtained as part of routine clinical care. We used multivariable Cox proportional hazards regression to examine the association between BP VIM during hospitalization and the development of incident dementia, determined by new ICD-9/10 coding or the new prescription of dementia medication, occurring at least 2 years after the index hospitalization.

**Results:**

Of 81,892 adults hospitalized without a prior ADRD diagnosis, 2,442 (2.98%) went on to develop ADRD (2.6 to 5.2 years after hospitalization). In multivariable-adjusted Cox models, both systolic (HR 1.05, 95% CI 1.00–1.09) and diastolic (1.06, 1.02–1.10) VIM were associated with incident ADRD. In pre-specified stratified analyses, the VIM associations with incident ADRD were most pronounced in individuals over age 60 years and among those with renal disease or hypertension. Results were similar when repeated to include incident ADRD diagnoses made at least 1 or 3 years after index hospitalization.

**Discussion:**

We found that measurements of BPV from acute care hospitalizations can be used to identify individuals at risk for developing a diagnosis of ADRD within approximately 5 years. Use of the readily accessible BPV measure may allow healthcare systems to risk stratify patients during periods of intense patient-provider interaction and, in turn, facilitate engagement in ADRD screening programs.

## Introduction

The growing burden of Alzheimer's disease and Related Dementias (ADRD) has prompted renewed calls for improved prognostic tools to help identify at risk patients prior to the development of clinical disease, when interventions to mitigate or slow cognitive decline may be more efficacious (1). The risk of ADRD increases following acute care hospitalization, indicating that focusing efforts on risk stratification of hospitalized patients may identify individuals who are more likely to go on to develop cognitive impairment (2, 3). While multiple screening modalities including cognitive testing, neurologic imaging, and even novel biomarkers have been developed, the feasibility of deploying these at scale among all patients is limited (4–10).

Conversely, hypertension represents a well-recognized and easily accessible ADRD risk factor readily assessed at a population level (11). Unfortunately, due to acute illness, pain, and medications, mean blood pressure assessment during acute hospitalizations does not necessarily reflect either hypertensive status or the severity of hypertension in the outpatient setting (12). Beyond the degree of blood pressure elevation, however, blood pressure variability (BPV) has also been linked to ADRD risk, with a recent meta-analysis indicating that the relative association between BPV and ADRD may be stronger than that of mean blood pressure alone (13, 14). While numerous cohort studies have evaluated the association of intermediate and long-term BPV with ADRD, little is known regarding potential association with short-term, acute BPV during hospital admission and subsequent ADRD diagnoses (15–20). Given that readily available and granular BPV data can be collected and analyzed from a hospitalization, often involving patients with at least age-based risk for ADRD, we sought to determine whether BPV measurements generated in this particular clinical setting could be useful for estimating risk for subsequent incidence of ADRD.

## Methods

### Study design and data extraction

We identified all patients admitted to Cedars-Sinai Medical Center (CSMC) between 2013 and 2019; Cedars-Sinai is a large, quaternary care facility located in Southern California, with a catchment area that includes a diverse population of 1.8 million individuals. We extracted demographics (age, sex, race/ethnicity), smoking status, and clinical comorbidities (diabetes, coronary artery disease, transient ischemic attack, stroke, hypertension, depression, atrial fibrillation or flutter, and concussion) using International Classification of Diseases (ICD)-9 and ICD-10 codes (Supplementary Table 1) at the time of hospitalization during the study period. We then extracted all inpatient systolic and diastolic blood pressure measurements from the hospitalization and calculated variability independent of the mean (VIM), as described previously (21).

### Variability independent of the mean calculation

Briefly, VIM is calculated first as the standard deviation of BP readings divided by the mean BP raised to the power of *x*, where *x* is obtained from fitting a non-linear regression model among the entire sample where standard deviation = a^*^mean^*x*^. This quantity is then multiplied by the sample mean BP raised to the power of *x*. Other methods of quantifying BPV such as standard deviation, coefficient of variation, and mean real variability are highly correlated with mean blood pressuring, limiting their utility in differentiating the effect of BPV from mean blood pressure. VIM was chosen for this study given its independence from mean blood pressure, allowing for decoupling of effects of mean blood pressure on BPV (22, 23).

### Statistical analysis

Our primary outcome was new dementia diagnosis, which we defined as the presence of ICD-9 or ICD-10 codes for dementia or the initiation of dementia medication (Supplementary Table 1). Analytical models were constructed to account for key clinical variables prioritized within a conceptual framework linking BPV with ADRD risk (Supplementary Figure 1) that is based on previously published evidence (14, 16, 24). We used multivariable Cox proportional hazards regression to examine the association between BP VIM during hospitalization and the development of incident dementia during follow-up. Cox models were adjusted for age, sex, race/ethnicity, smoking status, hospital length of stay, ICU status during hospitalization, number of BP readings, mean systolic and diastolic BP, and all extracted clinical comorbidities.

Given that dementia diagnoses tend to occur late in the onset of the disease, we assumed that a patient's BP VIM during hospitalization could only be associated with a captured diagnosis of dementia that occurred at least 2 years after the end of the hospitalization (25, 26). Therefore, only hospitalizations with at least 2 years of event-free follow-up (blanking period) were included in the analysis. In sensitivity analyses, we altered the follow-up periods to either 1 year or 3 years after hospital discharge. Follow-up time was defined as time from discharge until dementia diagnosis, death, or latest recorded outpatient visit up to December 31, 2019. For patients with multiple qualifying hospitalizations, BP VIM and follow-up time were calculated using the most recent hospitalization. We excluded patients with no qualifying hospitalizations, no recorded outpatient visits, or who were aged <18 years at the time of index hospitalization. We conducted all statistical analyses using R (v4.1.1) and considered statistical significance as a two-tailed *P*-value < 0.05.

### Standard protocol approvals, registrations, and patient consents

Study procedures were reviewed and approved by the Cedars-Sinai institutional review board (Study 00000603), with a waiver for informed consent.

## Results

We identified a total of *n* = 81,892 hospitalized patients without prior ADRD meeting follow up criteria, with an average age of 55.9 ± 18.8 years at the time of admission, of whom 50,808 (62.0%) were female. The median hospital length of stay was 65.7 (43.3, 103.6) hours, with 7,372 (9.0%) of patients admitted to the intensive care unit during their hospitalization. The most common clinical comorbidity was hypertension (29.0%), followed by depression (12.1%), and coronary artery disease (11.5%). The mean systolic and diastolic blood pressures during hospital stays were 122.4 ± 14.3 mmHg and 68.4 ± 9.2 mmHg, respectively, with a median of 40 (16, 43) BP measurement per patient. A total of 2,442 (3.0%) individuals developed incident ADRD at least 2 years following hospital discharge (Table 1); follow up of the total cohort ranged from 2.6 to 5.2 years, with a mean follow up of 3.6 years.

**Table 1:** Demographic and clinical characteristics of cohort, stratified by sex.

**Characteristic**	**Overall** **(*n =* 81892)**	**Female** **(*n =* 50808)**	**Male** **(*n =* 31084)**
**Demographic characteristics**			
Age, years, mean (SD)	55.88 (18.84)	52.55 (19.36)	61.33 (16.58)
Race/ethnicity, *n* (%)			
Asian	5744 (7.0)	4199 (8.3)	1545 (5.0)
Hispanic/Latinx	10346 (12.6)	6804 (13.4)	3542 (11.4)
Non-Hispanic Black	10631 (13.0)	6968 (13.7)	3663 (11.8)
Non-Hispanic White	51366 (62.7)	30404 (59.8)	20962 (67.4)
Other	3451 (4.2)	2222 (4.4)	1229 (4.0)
Unknown	354 (0.4)	211 (0.4)	143 (0.5)
Smoking status, *n* (%)			
Current	4597 (5.6)	1961 (3.9)	2636 (8.5)
Former	18353 (22.4)	9418 (18.5)	8935 (28.7)
Never	58942 (72.0)	39429 (77.6)	19513 (62.8)
**Clinical characteristics**			
ICU stay during index hospitalization, *n* (%)	7372 (9.0)	2919 (5.7)	4453 (14.3)
Length of index hospital length stay, mean (SD), hour	65.72 [43.33, 103.58]	65.5 [46.07, 98.59]	66.23 [37.35, 119.04]
Diabetes mellitus, *n* (%)	8065 (9.8)	3837 (7.6)	4228 (13.6)
Coronary artery disease, *n* (%)	9449 (11.5)	3153 (6.2)	6296 (20.3)
Atrial fibrillation or atrial flutter, *n* (%)	6644 (8.1)	2774 (5.5)	3870 (12.5)
Stroke, *n* (%)	5972 (7.3)	2894 (5.7)	3078 (9.9)
Renal disease, *n* (%)	7580 (9.3)	3074 (6.1)	4506 (14.5)
Hypertension, *n* (%)	23756 (29.0)	12010 (23.6)	11746 (37.8)
Depression, *n* (%)	9923 (12.1)	6763 (13.3)	3160 (10.2)
Transient ischemic attack, *n* (%)	3017 (3.7)	1541 (3.0)	1476 (4.7)
Concussion, *n* (%)	3256 (4.0)	1908 (3.8)	1348 (4.3)
**Blood pressure characteristics**			
Number of blood pressures recorded during hospitalization, mean (SD)	40.45 (69.81)	38.14 (55.71)	44.21 (88.00)
Number of blood pressures recorded during hospitalization, median (IQR)	25.00 [16.00, 43.00]	27.00 [17.00, 43.00]	23.00 [14.00, 43.00]
Mean systolic blood pressure, mean (SD), mmHg	122.36 (14.32)	120.41 (14.11)	125.54 (14.08)
Mean diastolic blood pressure, mean (SD), mmHg	68.36 (9.24)	66.62 (8.56)	71.20 (9.61)
Systolic variation independent of the mean, mean (SD)	11.95 (3.91)	12.02 (3.80)	11.83 (4.08)
Diastolic variation independent of the mean, mean (SD)	8.58 (2.74)	8.68 (2.73)	8.42 (2.74)

In multivariable analysis, greater inpatient systolic (Hazards Ratio 1.05, 95% CI 1.00–1.09) and diastolic (1.06, 1.02–1.10) VIM were each associated with increased risk of incident dementia at least 2 years following hospitalization (Table 2). As expected, greater mean systolic BP was also associated with increased ADRD risk (1.01 per 1 mmHg increase in systolic BP, 1.01–1.01). In sex stratified analyses, only diastolic VIM among females remained significantly associated with incident ADRD (1.07, 1.02–1.13), though no differences in risk by sex was appreciated.

**Table 2:** Risk of incident ADRD diagnosis in the 2 years following an acute care hospitalization, overall and stratified by sex.

	**Overall (*****n =*** **81892)**		**Female (*n =* 50808)**	**Male (*n =* 31084)**	***p*** **-value^2^**
	**Crude HR** **(95% CI)**	**Adjusted HR** **(95% CI)**^1^	**Adjusted HR** **(95% CI)**^1^	**Adjusted HR** **(95% CI)**^1^	
Systolic VIM	1.39 (1.34, 1.44)	**1.05 (1.00, 1.09)**	1.05 (1.00, 1.11)	1.05 (0.99, 1.11)	0.909
Diastolic VIM	1.31 (1.27, 1.36)	**1.06 (1.02, 1.10)**	**1.07 (1.02, 1.13)**	1.05 (0.99, 1.12)	0.564
Incident dementia, *n* (%)		2442 (2.98%)	1324 (2.61%)	1118 (3.6%)	

CI, confidence interval; HR, hazard ratio; VIM, variation independent of the mean.

1 Cox models adjusted for age, sex, race/ethnicity, smoking status, ICU stay during index hospitalization, number of blood pressure measurements, mean systolic and diastolic blood pressure, length of hospital stay, diabetes mellitus, coronary artery disease, stroke, hypertension, depression, transient ischemic attack, and concussion.

2 P-values for sex interaction, i.e., difference in adjusted HRs between males and females. Bold indicates statistically significant adjusted values.

**Figure 1:**
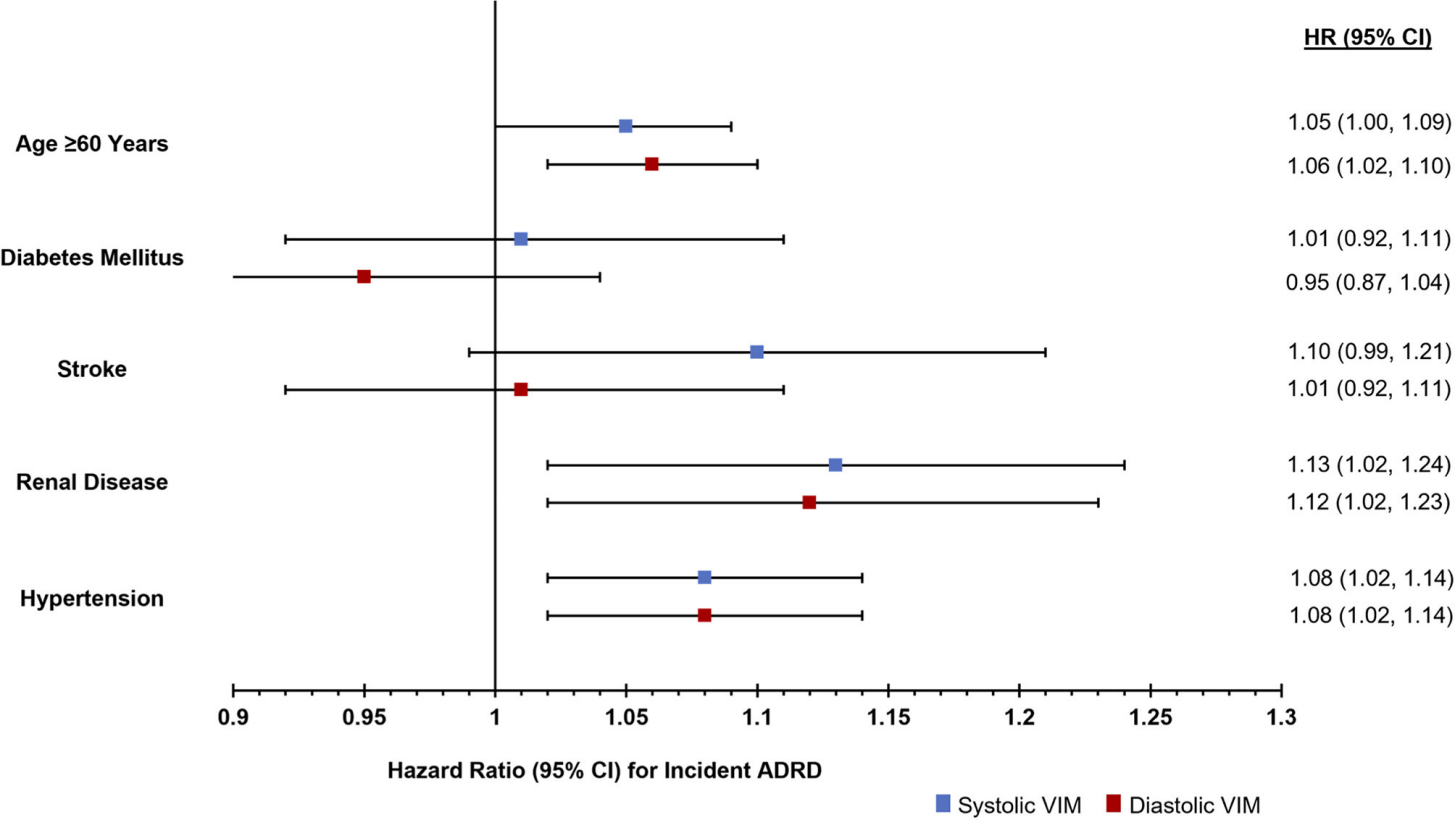
Risk of incident ADRD diagnosis in the 2 years following an acute care hospitalization stratified by comorbidities.

Stratification by clinical characteristics demonstrated significant associations between higher systolic and diastolic VIM among individuals over the age of 60 at the time of hospitalization, but not those ≤60 years of age. Greater systolic and diastolic VIM were also associated with ADRD among those with renal disease and those with hypertension, but not those with stroke or diabetes (Figure 1; Supplementary Table 2).

In sensitivity analyses, a total of 106,000 individuals had qualifying hospitalizations with at least 1 year of follow up data, of whom 3,437 (3.2%) developed incident dementia at least 1 year after hospitalization. Greater systolic (1.08, 1.04–1.11) and diastolic (1.06, 1.03–1.09) VIM were each associated with increased ADRD during the follow up period, a finding that was robust to stratification by sex, without differences appreciated between women and men. Conversely, a total of 62,615 individuals had qualifying hospitalizations with at least 3 years of follow up data, of whom 1,683 (2.7%) developed incident dementia at least 3 years after hospitalization. Greater diastolic (1.06, 1.03–1.09), but not systolic (1.03, 0.98–1.08) VIM was associated with increased ADRD during the follow up period. In sex stratified analyses, diastolic VIM remained significant only for females, but without a detectable difference between sexes (Supplementary Table 3).

## Discussion

In a large cohort study of over 80,000 hospitalized individuals, we found that greater BPV, derived from clinically generated data during hospital admission, is associated with incident ADRD diagnosis at least 2 years after acute care hospitalization. This association held for both systolic and diastolic VIM, with robust diastolic VIM findings to variation in the duration of follow up. The utilization of routinely captured, readily accessible, and quantifiable data to assist in patient and population level ADRD risk assessment, represents a novel and effective approach to appropriately target further testing and treatment to high-risk individuals. In the future, we anticipate inpatient BPV serving as a risk marker that may prompt clinicians to pursue diagnostic ADRD testing.

Worldwide an estimated 46.8 million people are afflicted with dementia, with rates expected to triple by 2050 due to increasing life expectancy (27). Due in part to high failure rates of clinical trials aimed at Alzheimer's disease modification, in recent years there has been increased emphasis on risk stratification and risk reduction (28). Midlife vascular risk factors, including hypertension, have been associated with elevated brain amyloid deposition and an increased risk of developing dementia (29). While antihypertensive therapy especially in midlife represents a potentially viable intervention to reduce risk for dementia, there are inconsistencies on optimal blood pressure targets and uncertainties on the choice of antihypertensive agents (30). Visit-to-visit intra individual BPV in the outpatient setting has been associated with higher risk of dementia and cognitive impairment and may provide an alternate link to blood pressure and dementia risk (14). To our knowledge, there has not been a study that examined BPV in the acute hospital setting and the risk for dementia.

Our findings address two important gaps in current risk prediction methodologies. First, the use of routinely generated clinical data, easily accessible from the electronic health record (EHR), assists in reducing the burden of large scale ADRD screening volumes. By identifying patients at high-risk for incident or undiagnosed ADRD, screening can be targeted to an enriched population most likely to benefit. Other well recognized, easily accessible clinical variables indicative of increased ADRD risk such as advanced age and high blood pressure are exceedingly common, providing poor discrimination for truly high-risk individuals. Importantly, BP data obtained in cohort studies are typically captured using high-fidelity protocols that are not often used in clinical practice (31–34). Our results demonstrate a preserved signal of BPV associated ADRD risk in clinically generated data, including BP measures obtained using different techniques and devices as is the norm in practice, opening the possibility of using this metric in real-world patient care settings. Second, the use of data solely from an acute hospitalization creates an opportunity to provide risk assessment for individuals without care continuity in a single healthcare system. The disjointed nature of US healthcare systems may preclude any single organization's ability to use outpatient VIM to predict ADRD risk. Further, some patients are unable or choose not to obtain consistent care from a single system, hindering clinicians' ability to effectively track, identify and manage early signs of ADRD. Inpatient hospital stays offer a unique opportunity of high healthcare contact for patients during which otherwise unrecognized or untreated conditions can be addressed. High blood pressure variability may now help clinicians identify and screen patients for ADRD, either during hospitalization or through coordination of outpatient care. Sharing of positive screening results with patients may also spur greater engagement with the medical system, overcoming inertia that could otherwise delay diagnosis until later stages of disease (35).

In stratified analyses, we found that BPV's association with ADRD was particularly noted among those over the age of 60, as well as those with renal disease and a history of hypertension. These findings are not surprising given both the increased incidence of ADRD with advancing age, as well as the known associations between hypertension and both kidney disease and ADRD. Further linking these factors, the association between hypertension and ADRD is strongest for individuals in mid- to late-life (11). Screening efforts focused on patients with high BPV in this age range, particularly those with a history of hypertension or kidney disease may offer the most efficacious use of limited screening capacity. In the absence of any sex differences for systolic VIM, sex-stratified analyses suggested that diastolic VIM may be associated with ADRD in women and not in men. This finding could be related to females having smaller caliber arteries than males, even after adjusting for body size, (36) and thus a greater end-organ sensitivity to fluctuations in basal hemodynamic pressure; (37) additional studies are needed to further investigate and validate these findings.

The biological underpinnings of the association of BPV and ADRD remains unclear. Vascular dysfunction and damage represent pathophysiologic mechanisms of cognitive impairment which can occur acutely, as with stroke, or chronically, as with ADRD (38–40). Greater BPV has been linked to increased vascular dysfunction, providing a potential mechanism of BPV leading to future ADRD. Such a relationship would indicate that reducing BPV, through either pharmacologic or non-pharmacologic mechanisms, may result in reductions in vascular disease and subsequent ADRD diagnosis or disease progression. Conversely, autonomic dysfunction has long been a recognized sequela of ADRD, with patients suffering from orthostatic hypotension, heart rate variability, and constipation (41, 42). In this setting, BPV may represent a ‘canary in the coalmine' of early ADRD, signaling future cognitive dysfunction before neurologic effects become clinically relevant (43–45). In this situation, BPV may represent both an early warning sign for future ADRD, as well as a potential surrogate marker for efficacy of disease management.

### Limitations

Several limitations of this study merit consideration. First, the retrospective nature of the analysis clearly prohibits causal conclusions. As detailed above, however, the identification of BPV and ADRD using real-world data, may still prove clinically useful, as the purpose if one of risk identification, rather than demonstrating causality. Further, the analysis relies on administrative coding for identification of the outcome and patient level comorbidities. Fortunately, these codes have been well studied in other analysis and demonstrated the ability to appropriately identify patients with the specified conditions (46–48). Along these lines, other recognized ADRD risk factors including family history of dementia, physical activity, and educational attainment could not be accurately extracted from the EHR, and as such, were not included in the model, potentially confounding our results (49, 50). Prescription of antihypertensive medications was also not included in our models as these medications are typically discontinued or otherwise adjusted during hospitalization. Again, given the goal of identifying a marker of ADRD risk using data that is readily available at a population level in real work settings, the results still point to high inpatient VIM as a potential mechanism for identifying high-risk patients at the point of care.

## Conclusion

The derivation of BPV using clinically generated blood pressure measurements during acute care hospitalizations can be used to identify individuals at high-risk for the development of ADRD in the short to intermediate time horizon. The use of this marker may allow healthcare systems to risk stratify patients at a population level during a time of high patient-provider interaction to engage patients in ADRD screening programs.

## Data availability statement

The datasets presented in this article are not readily available due to the sensitive nature of the data collected for this study. Requests to access the dataset from qualified researchers trained in protocols on the protection of human subjects may be sent to Cedars-Sinai Medical Center at biodatacore@cshs.org.

## Ethics statement

The studies involving human participants were reviewed and approved by the Cedars-Sinai Institutional Review Board. Written informed consent for participation was not required for this study in accordance with the national legislation and the institutional requirements.

## Author contributions

Co-authors JE, PB, SC, and ZT were responsible for the acquisition of data. JE, SC, and ZT were responsible for the study concept/design. JE, MD, MW, SC, and ZT were responsible for the analysis or interpretation of data. All authors contributed to the article and approved the submitted version.
