# Supplementary material for: Association of blood pressure variability during acute care hospitalization and incident dementia
Source: Front Neurol. 2023 Feb 7;14:1085885. doi: 10.3389/fneur.2023.1085885 (PMC9941567; doi:10.3389/fneur.2023.1085885)
Supplement: Supplementary file 1 [file Data_Sheet_1.docx]

**Association of Blood Pressure Variability During Acute Care Hospitalization and Incident Dementia**

Supplemental Information

Supplemental Figure 1 …. Pg 2

Supplemental Table 1 …. Pg 3

Supplemental Table 2 …. Pg 4

Supplemental Table 3 …. Pg 5

**Correspondence.** Joseph E. Ebinger, MD, MS, Department of Cardiology, Smidt Heart Institute, Cedars-Sinai Medical Center, Los Angeles, CA, USA, Phone (310) 423-2726**,** Email [joseph.ebinger@csmc.edu](mailto:joseph.ebinger@csmc.edu)

**
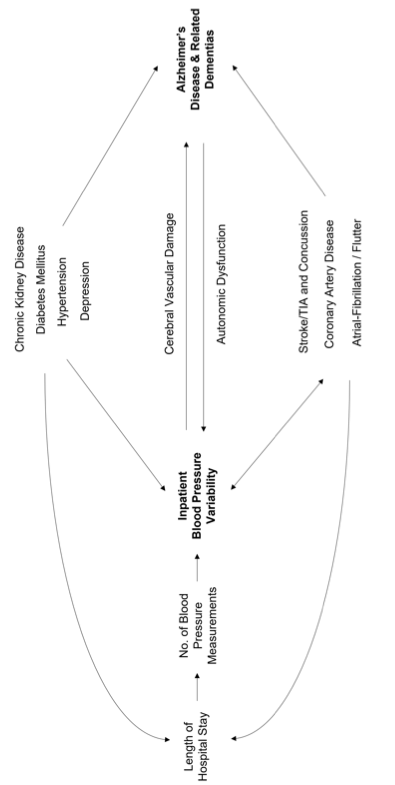
Supplemental Figure 1.** Conceptual framework linking blood pressure variability and Alzheimer’s Disease and Related Dementia risk.

**Supplemental Table 1.** Administrative codes and medications for the identification of Alzheimer’s Disease and Related Dementias and pre-specified clinical comorbidities of interest.

|  | **ICD-9 and ICD-10 Codes** | **Medications** |
| --- | --- | --- |
| **Outcome** |  |  |
| Alzheimer’s Disease and Related Dementias | 46.1*, 291.2, 294.1*, 294.2*, 290.4*, 331.0-331.2, 331.4, 331.82, 332.0, 333.0, 797, A81.0*, F02.8*, F03.9*, F01.5*, F10.27, G31.0*, G31.1, G31.83, R41.81, G20, G23.1, G30.* | Aricept, Namzaric, Exelon, Razadyne, Reminyl, Cognex, Memantine |
| **Comorbid Conditions** |  |  |
| Coronary Artery Disease | 414.11, 414.12, 414.2, I25.1*, I25.7*, I25.8*, 411.1, 411.81, I25.4, I25.10, 414.0* |  |
| Diabetes Mellitus | 250.*, E10.*, E11.*, E13.*, E14.* |  |
| Atrial Fibrillation/Atrial Flutter | 427.31, 427.32, I48.0*, I48.1*, I48.2*, I48.3*, I48.4*, I48.9* |  |
| Hypertension | 403.00, 403.01, 403.10, 403.11, 403.90, 403.91, 404.*, 582.*, 585.*,586.*, 588.0, I12.0, I12.9, I13.0, I13.1, I13.10, I13.11, I13.2, I13.9, N25.0 Z49.0, Z49.1, Z49.2, Z94.0, Z99.2, 583.*, N03.*, N04.*, N05.*, N18*,N19* |  |
| Stroke | 362.3*, 430.*, 431.*, 433.*, 434.*, G46.*, I60.*, I61.*, I62.*, I63.*, I64.*, I65.*, I66.*, I67.*, S06.5 |  |
| Transient Ischemic Attack | 435.*, G45.0, G45.1, G45.8, G45.9, I67.848 |  |
| Concussion, Head Trauma, Traumatic Brain Injury | 959.01, 850.9, 854.0, S06.0X9A, S06.9*, S09.90 |  |
| Depression and Anxiety | 296.2*, 296.3*, 625.4, 293.83, 311, 648.4*, 293.89, 300*, F32.0-F32.5, F32.81, F32.9, F33*, F33.41, F33.42, N94.3, F06.30, O99.34*, F53*, F41.1-F41.9 |  |

**Supplemental Table 2.** Risk of incident ADRD diagnosis in the 2 years following an acute care hospitalization stratified by comorbidities.

|  | **Age** | | **p-value^2^** | **Diabetes** | | **p-value^2^** | **Stroke** | | **p-value^2^** | **Renal disease** | | **p-value^2^** | **Hypertension** | | **p-value^2^** |
| --- | --- | --- | --- | --- | --- | --- | --- | --- | --- | --- | --- | --- | --- | --- | --- |
|  | **≥60 yrs**  **(N=37181)** | **<60 yrs**  **(N=44711)** |  | **Yes (N=8065)** | **No (N=73827)** |  | **Yes (N=5972)** | **No (N=75920)** |  | **Yes (N=7580)** | **No (N=74312)** |  | **Yes (n=23756)** | **No (n=58136)** |  |
|  | *Adjusted HR*  *(95% CI)^1^* | *Adjusted HR*  *(95% CI)^1^* |  | *Adjusted HR*  *(95% CI)^1^* | *Adjusted HR*  *(95% CI)^1^* |  | *Adjusted HR*  *(95% CI)^1^* | *Adjusted HR*  *(95% CI)^1^* |  | *Adjusted HR*  *(95% CI)^1^* | *Adjusted HR*  *(95% CI)^1^* |  | *Adjusted HR*  *(95% CI)^1^* | *Adjusted HR*  *(95% CI)^1^* |  |
|  |  |  |  |  |  |  |  |  |  |  |  |  |  |  |  |
| **Systolic VIM** | **1.05 (1.00, 1.09)** | 1.05 (0.90, 1.22) | 0.79 | 1.01 (0.92, 1.11) | **1.05 (1.01, 1.10)** | 0.44 | 1.10 (0.99, 1.21) | 1.04 (0.99, 1.08) | 0.31 | **1.13 (1.02, 1.24)** | 1.03 (0.98, 1.07) | 0.08 | **1.08 (1.02, 1.14)** | 1.02 (0.96, 1.08) | 0.20 |
| **Diastolic VIM** | **1.06 (1.02, 1.10)** | 1.06 (0.92, 1.23) | 0.98 | 0.95 (0.87, 1.04) | **1.09 (1.04, 1.13)** | **0.011** | 1.01 (0.92, 1.11) | **1.07 (1.03, 1.12)** | 0.28 | **1.12 (1.02, 1.23)** | **1.05 (1.01, 1.09)** | 0.18 | **1.08 (1.02, 1.14)** | 1.04 (0.99, 1.10) | 0.34 |
|  |  |  |  |  |  |  |  |  |  |  |  |  |  |  |  |
| **Incident Dementia, n (%)** | 2234 (6.01%) | 208 (0.47%) |  | 408 (5.06%) | 2034 (2.76%) |  | 369 (6.18%) | 2073 (2.73%) |  | 338 (4.46%) | 2104 (2.83%) |  | 1118 (4.71%) | 1324 (2.28%) |  |
|  |  |  |  |  |  |  |  |  |  |  |  |  |  |  |  |
| *Abbreviations: CI, confidence interval; HR, hazard ratio; VIM: variation independent of the mean*  1. Cox models adjusted for age, sex, race/ethnicity, smoking status, ICU stay during index hospitalization, number of blood pressure measurements, mean systolic and diastolic blood pressure, length of hospital stay, diabetes mellitus, coronary artery disease, stroke, hypertension, depression, transient ischemic attack, and concussion. Models exclude patients with unknown race/ethnicity due to model convergence issues.  2. P-values for subgroup interaction, e.g. difference in adjusted HRs between diabetes vs. no diabetes, etc. | | | | | | | | | | | | | | | |

**Supplemental Table 3.** Sensitivity analysis of risk of incident ADRD diagnosis following an acute care hospitalization, overall and stratified by sex, with A) 1-year and B) 3-year blanking periods.

1. 1-year blanking period

| **Outcome** | **Overall (n=106000)** | | **Female (n=64566)** | **Male (n=41434)** | **p-value^2^** |
| --- | --- | --- | --- | --- | --- |
|  | *Crude HR (95% CI)* | *Adjusted HR (95% CI)^1^* | *Adjusted HR (95% CI)^1^* | *Adjusted HR (95% CI)^1^* |  |
|  |  |  |  |  |  |
| Systolic VIM | 1.42 (1.38, 1.47) | **1.08 (1.04, 1.11)** | **1.09 (1.04, 1.14)** | **1.07 (1.02, 1.12)** | 0.570 |
| Diastolic VIM | 1.32 (1.28, 1.36) | **1.06 (1.03, 1.09)** | **1.04 (1.00, 1.08)** | **1.10 (1.05, 1.15)** | 0.094 |
|  |  |  |  |  |  |

1. 3-year blanking period

| **Outcome** | **Overall (n=62615)** | | **Female (n=39111)** | **Male (n=23504)** | **p-value^2^** |
| --- | --- | --- | --- | --- | --- |
|  | *Crude HR (95% CI)* | *Adjusted HR (95% CI)^1^* | *Adjusted HR (95% CI)^1^* | *Adjusted HR (95% CI)^1^* |  |
|  |  |  |  |  |  |
| Systolic VIM | 1.36 (1.30, 1.42) | 1.03 (0.98, 1.08) | 1.03 (0.96, 1.10) | 1.04 (0.97, 1.12) | 0.869 |
| Diastolic VIM | 1.29 (1.24, 1.35) | **1.06 (1.01, 1.11)** | **1.07 (1.01, 1.14)** | 1.06 (0.98, 1.13) | 0.712 |
|  |  |  |  |  |  |

Abbreviations: CI, confidence interval; HR, hazard ratio; VIM: variation independent of the mean

1. Cox models adjusted for age, sex, race/ethnicity, smoking status, ICU stay during index hospitalization, number of blood pressure measurements, mean systolic and diastolic blood pressure, length of hospital stay, diabetes mellitus, coronary artery disease, stroke, hypertension, depression, transient ischemic attack, and concussion.

2. P-values for sex interaction, i.e. difference in adjusted HRs between males and females.
